# Case Report: Serial MR Vessel Wall Imaging Visualizes the Response of Intracranial Plaques and Assists in Decision-Making

**DOI:** 10.3389/fnins.2021.739178

**Published:** 2021-10-20

**Authors:** Jiayu Xiao, Matthew M. Padrick, Shlee S. Song, Zhaoyang Fan, Konrad H. Schlick

**Affiliations:** ^1^Departments of Radiology, Keck School of Medicine of USC, Los Angeles, CA, United States; ^2^Biomedical Imaging Research Institute, Department of Biomedical Sciences, Cedars-Sinai Medical Center, Los Angeles, CA, United States; ^3^Department of Neurology, Cedars-Sinai Medical Center, Los Angeles, CA, United States; ^4^Departments of Radiation Oncology, Keck School of Medicine of USC, Los Angeles, CA, United States

**Keywords:** intracranial atherosclerosis, stroke, plaque, vessel wall imaging, follow-up study

## Abstract

Intracranial atherosclerotic disease (ICAD) is a dynamic process that leads to ischemic stroke. Symptomatic ICAD patients still suffer a high recurrent rate even under standard treatment. In this case report, to better understand the response of intracranial atherosclerotic plaques to medication, serial MR imaging was added to standard clinical workup in a 47-year-old male patient with acute occipital lobe infarction at baseline, 3-month, 6-month, and 12-month post index stroke to directly visualize the morphology and signal change of plaques. We noticed that one of the plaques showed dramatic worsening at 3-month imaging follow-up despite a decrease in low-density lipoprotein level. Early identification of patients who do not respond well to medication is critical to prevent the recurrence of cardiovascular events in ICAD patients.

## Introduction

Intracranial atherosclerotic disease (ICAD) represents a significant risk factor for ischemic stroke worldwide, and patients with symptomatic ICAD experience a high rate of recurrent stroke (Holmstedt et al., 2013). Therefore, active monitoring of intracranial atherosclerotic lesions has been crucial for the secondary prevention of stroke. Current clinical practice relies on physical examinations, laboratory tests, and conventional imaging modalities like CT angiography or MR angiography (MRA), which mainly focus on the assessment of luminal stenosis. In recent years, the value of magnetic resonance vessel wall imaging (MR-VWI) in directly characterizing the morphological features of intracranial atherosclerotic plaques has been explored (Song et al., 2021). In this case report, we used MR-VWI to directly probe atherosclerotic lesions in an effort to reveal lesion-level response to medical treatment.

## Case Description

A 47-year-old man presented with headache and vertigo. Before the index stroke event, the patient had been diagnosed with hyperlipidemia for more than 10 years without being regularly monitored or taking lipid-lowering medications. He had no history of hypertension, diabetes, or smoking. CT angiography reported a thrombosed right vertebral artery (VA) V4 segment with luminal irregularities, with concerns for focal dissection, atherosclerosis, or both. This prompted initiation of intravenous heparin. Routine brain MR revealed an acute occipital lobe infarction and a slight increase in pre-contrast T1-weighted signal within the right VA V4 segment. Other laboratory findings included low-density lipoprotein (LDL) of 123 mg/dL and hemoglobin A1c of 5.7%.

**Figure 1:**
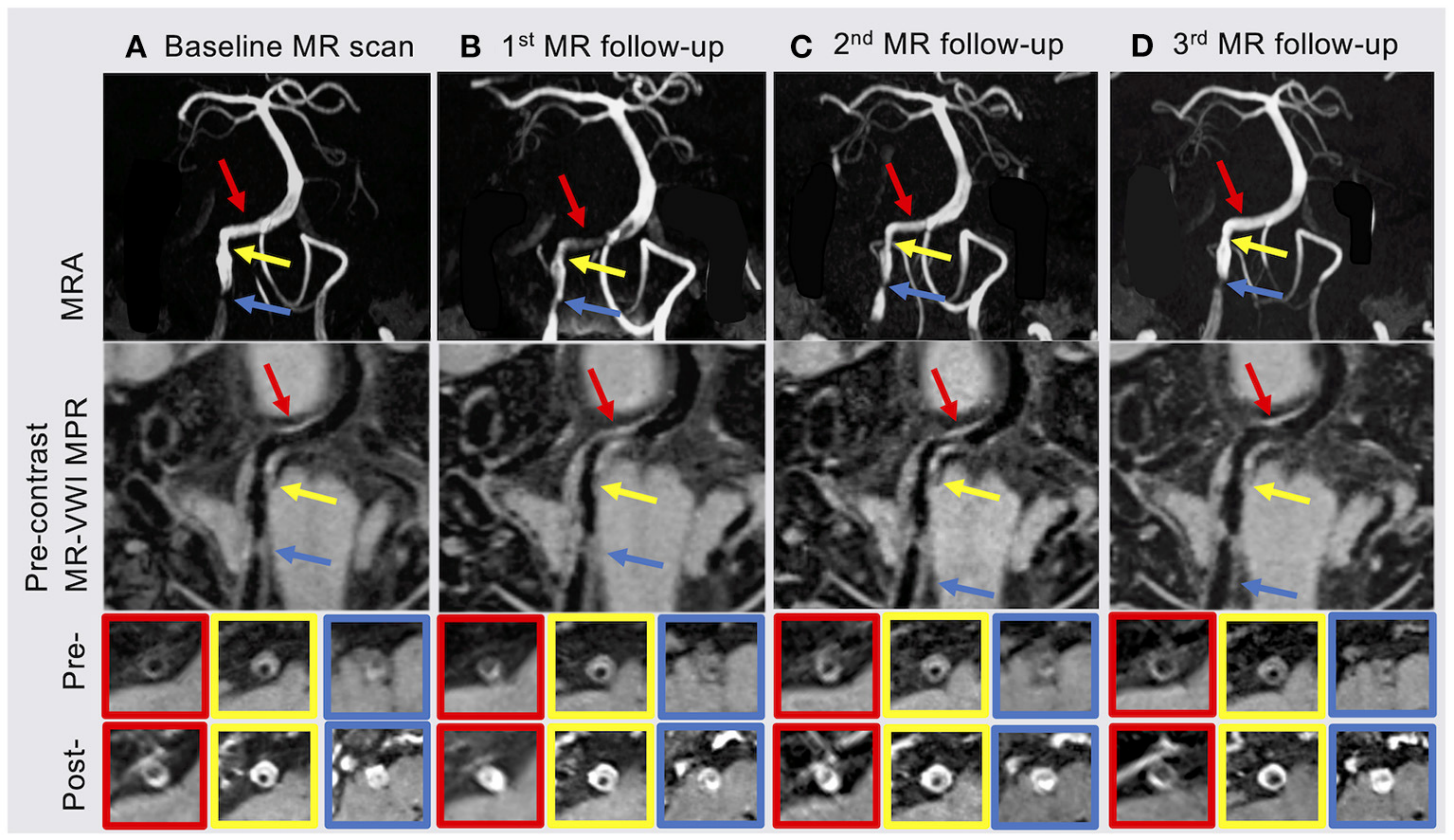
**(A)** Baseline MR scan showed two plaques in the proximal (blue arrow) and middle (yellow arrow) of the right vertebral artery (VA) V4 segment. **(B)** The patient underwent the 1st MR follow-up scan 3 months after index stroke. MRA and MR-VWI showed a new plaque (red arrow) in the distal right VA V4 segment with severe stenosis and high enhancement ratio (ER). **(C)** Most of plaque features showed slightly regression at the 2nd follow-up MR scan 6 months after index stroke. **(D)** The 3rd follow-up MR scan 12 months after index stroke showed that the degree of stenosis, plaque-wall contrast ratio (CR) and ER of the plaque in the distal right VA V4 segment were back to baseline and slightly improved in the other two plaques. MRA, MR angiography; MR-VWI, MR vessel wall imaging; MPR, multiplanar reconstruction; Pre-, pre-contrast MR-VWI; Post-, post-contrast MR-VWI.

Due to the uncertainty involved with the lesion on the right V4 segment, further neurovascular imaging including 3D time-of-flight MRA and pre- and post-contrast MR-VWI (Fan et al., 2017; Yang et al., 2017) was pursued. MRA revealed two stenotic lesions in the right VA (Figure 1 yellow and blue arrows). Eccentric wall thickening, presence of hyper-intensity on pre-contrast MR-VWI and strong contrast enhancement on post-contrast MR-VWI highly suggested atherosclerotic disease rather than dissection. We quantified the degree of stenosis, pre-contrast plaque-wall contrast ratio (CR), and post-contrast plaque enhancement ratio (ER) at individual lesions using previously published methods (Wu et al., 2018). Specifically, 3D MR-VWI images were reformatted to cross-sectional views, and mean signal intensity was quantified with manually drawn regions of interest. Plaque-wall CR was calculated as the signal intensity ratio between the brightest region within the plaque and the adjacent normal-looking vessel wall. Plaque ER was calculated as the normalized signal intensity ratio between the post-contrast plaque and pre-contrast plaque. Reliability of these quantitative measurements was excellent in patients with ICAD as shown in a recent paper (Zhang et al., 2021). In the meantime, an asymptomatic plaque was also observed in the proximal V4 segment of the left vertebral artery. While in this patient, the left vertebral artery did not converge with the right vertebral artery to form the basilar artery. Quantitative analyses were not performed on this plaque because no significant changes were visualized.

High dose statin (atorvastatin 80 mg daily) and dual antiplatelet therapy (aspirin 325 mg daily and clopidogrel 75 mg daily) was initiated. The patient was hospitalized for 5 days. He was discharged with a minor headache and a National Institutes of Health Stroke Scale score of 0. Two months later, direct LDL level showed a decrease to 95 mg/dL at clinical follow-up. There were no adverse effects from the statin therapy noted. At this point, ezetimibe 10 mg daily was initiated by his cardiologist for further lowering the LDL level. However, statin therapy was stopped thereafter due to miscommunication between the patient and cardiologist.

At 3 months after the index stroke, a follow-up MR scan was performed. Despite the moderate regression of LDL and an appreciable drop in plaque-wall CR and/or plaque ER in the two originally identified lesions, a new lesion in the distal right VA with a diffusive and severe lumen narrowing and strong vessel wall enhancement was observed (Figure 1 red arrow). The degree of stenosis of the distal lesion increased from 24.42 to 80.11% and ER increased from 2.20 to 2.59. Therefore, high dose statin was resumed.

At 6 months after the index stroke, the patient was unable to tolerate ezetimibe due to a persistent skin rash, thus was placed on the PCSK-9 inhibitor evolocumab (140 mg subcutaneously every 2 weeks), which led to a drastic reduction in LDL from 67 mg/dL at 6 months to 18 mg/dL at 11 months.

MRA and MR-VWI scans were performed at 6- and 12-month post index stroke. Compared with the 3-month scan, patient's 6-month MRA showed a slight regression of lumen at the plaque in the distal right VA, but plaque ER measured on MR-VWI substantially reduced. Twelve months after the index stroke, both degree of stenosis measured on MRA and plaque ER measured on MR-VWI regressed to the baseline level at the plaque in the distal right VA. CR and ER of the other two plaques slightly improved while the degree of stenosis remained unchanged. During this period, the patient's condition has been stable without any new symptoms.

**Figure 2:**
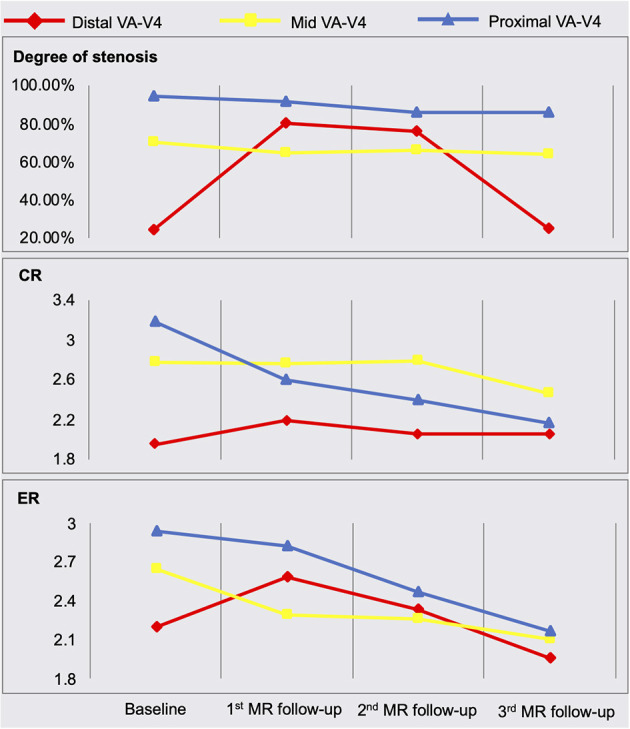
Quantitative measurement results of plaque features. MRA, MR angiography; MR-VWI, MR vessel wall imaging; VA, vertebral artery; CR, plaque-wall contrast ratio; ER, plaque enhancement ratio.

**Figure 3:**
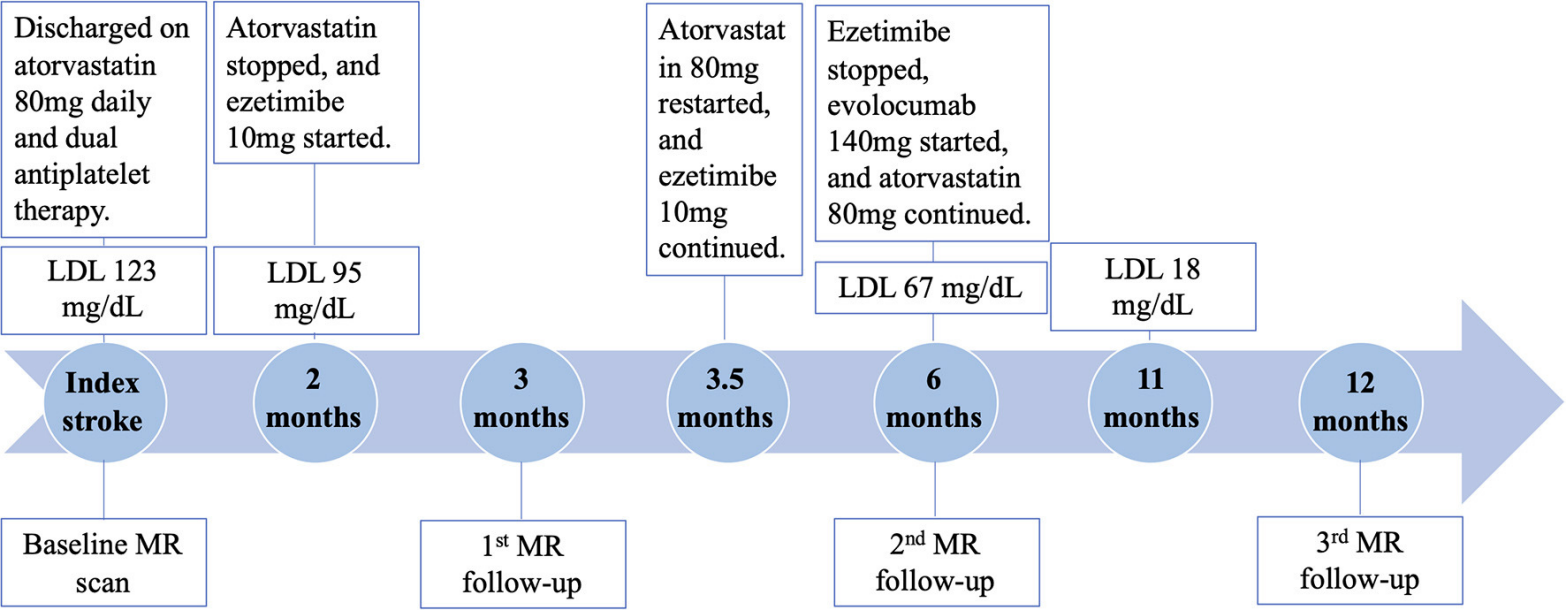
Timeline of clinical management and MR examinations.

The quantitative measurement results of plaque features are shown in Figure 2. A schematic diagram showing the time course of clinical management and MR examinations is presented in Figure 3.

## Discussion

Serum lipid biomarkers are widely accepted to monitor ICAD patients' responses to treatment; however, these are systemic rather than lesion-specific markers. MR-VWI can more specifically interrogate each atherosclerotic lesion and provide information beyond the severity of stenosis. Previous studies (Chen et al., 2018; Chung et al., 2020) focused on investigating the treatment effect of specific medical therapies using MR-VWI before and after 6-month treatment. In contrast, we performed serial MR-VWI at multiple time points (from 1 week after symptom onset through 1 year), with a focus on demonstrating that serial MR-VWI may reveal lesion-level response or resistance during medical treatment, which may not be adequately captured by routine assessments such as laboratory test. Another noteworthy novelty in our study is that we used a 3D whole-brain rather than 2D MR-VWI approach, which allowed for the detection of new plaques at other locations and the quantification of the signal contrast between plaque and normal vessel wall. As seen in the baseline and 3 months follow-up MR scans, the subsequently identified lesion in the distal VA, although not recognized in MRA, showed considerably high plaque-wall CR and plaque ER in MR-VWI and rapidly developed severe stenosis, indicating its active atherosclerotic activities (Wu et al., 2018).

In our case, we monitored the patient's LDL level and reinforced lifestyle modifications as standard of care. Additionally, we monitored imaging features including the degree of stenosis, plaque-wall CR (which might be related to intraplaque hemorrhage), and plaque ER (a marker of inflammation) (Mandell et al., 2017). Despite a decrease in serum LDL, this patient's plaque showed dramatic worsening based on these imaging features at 3 months. The underlying reasons for this mismatch of controlled LDL and plaque-worsening remain unclear. A probable reason could be due to the fact that they probe the response or resistance from different aspects, i.e., one at a systematic level and the other at a lesion level. However, there might be other confounding factors that we are not aware of given the drastic deterioration in such a short time period. A large-scale prospective study is warranted to explore the incidence of mismatches between systemic blood biomarkers and plaque features, and the added value of MR-VWI in the management of symptomatic ICAD patients.

The LDL reduction and plaque rapid progression observed in this case illustrate how serial imaging examinations can complement systemic biomarkers and assist in the management of secondary stroke prevention. Due to miscommunication, atorvastatin was discontinued for 1 month, during which time, only ezetimibe was used for lipid-lowering. The discontinuation of atorvastatin could be found out and corrected by the treating physician at the 3-month clinical visit. However, the 3-month MR-VWI results did propel the physician to prescribe a more intensive therapy and target an LDL level <20 mg/dL as a recent study has shown that further lowering of LDL beyond the current target (100 mg/dL) would further reduce cardiovascular risk (Sabatine et al., 2018). This case involved a non-standard therapy. However, it is not uncommon to see in clinical practice that an originally prescribed standard treatment plan can be compromised due to miscommunication or poor compliance. In this representative case, our findings highly suggest that serial MR-VWI may early detect lesion-level response or resistance during medical treatment, and this information can be supplemental to routine assessments such as laboratory test in guiding treatment plans. A treatment plan may be modified in a timely manner to better achieve secondary stroke prevention (Xiao et al., 2021). Of course, large-scale clinical trials are warranted to validate this hypothesis.

Since treatment responses may differ in individuals due to different pharmacodynamics and treatment compliance, longitudinal assessment is necessary for ICAD. However, there is not enough evidence about the best follow-up interval. The patient underwent his follow-up imaging at 3, 6, and 12 months following our ongoing research protocol. Physicians typically follow up with stroke patients and make decisions on medical management at 3 months. Therefore, we chose to repeat imaging at 3 months to provide more information to clinical practice. In this case report, given the notable plaque progression seen on the first follow-up scan, we postulate that repeat MR-VWI as soon as 3 months post ischemic stroke may be reasonable.

Additionally, PSCK-9 inhibitor effect on ICAD plaques should be explored further, as there may be an underlying mechanism beyond plummeting serum LDL level. Further, the expected temporal concordance between LDL levels and MR-VWI features will need to be elucidated, if any are present.

In this work, plaque composition and its change process were not investigated. This is because we used an MR-VWI protocol that involves a T1-weighted sequence only. This protocol features relatively short scan time and capability of quantifying temporal changes associated with intraplaque hemorrhage and inflammation. However, certain important plaque components, such as fibrous cap and lipid core, cannot be detected because several reasons: (1) the small size of intracranial vessels, (2) the limited spatial resolution, (3) the lack of additional contrast weighting such as T2-weighting. A high-resolution multi-contrast protocol would make MR-VWI challenging for both patients and clinical workflow.

In summary, this case report sheds light on the importance of the promising combination of serum biomarkers and lesion-specific imaging tools in the decision-making of stroke patients.

## Data Availability Statement

The original contributions presented in the study are included in the article/supplementary material, further inquiries can be directed to the corresponding authors.

## Ethics Statement

The studies involving human participants were reviewed and approved by Ethics Committee of Cedars-Sinai Medical Center. The patients/participants provided their written informed consent to participate in this study.

## Author Contributions

JX and MP interpreted the data and drafted the manuscript. SS, ZF, and KS contributed substantially to the design of the work and revised it critically for important intellectual content. All authors contributed to the article and approved the submitted version.

## Funding

SS and ZF received salary support from the NIH/NHLBI grant # R01HL147355.

## Conflict of Interest

The authors declare that the research was conducted in the absence of any commercial or financial relationships that could be construed as a potential conflict of interest.

## Publisher's Note

All claims expressed in this article are solely those of the authors and do not necessarily represent those of their affiliated organizations, or those of the publisher, the editors and the reviewers. Any product that may be evaluated in this article, or claim that may be made by its manufacturer, is not guaranteed or endorsed by the publisher.
